# Modulation of Xenobiotic Receptors by Steroids

**DOI:** 10.3390/molecules18077389

**Published:** 2013-06-24

**Authors:** Monimoy Banerjee, Delira Robbins, Taosheng Chen

**Affiliations:** Department of Chemical Biology and Therapeutics, St. Jude Children’s Research Hospital, 262 Danny Thomas Place, Memphis, TN 38105, USA

**Keywords:** nuclear receptors, pregnane X receptor: constitutive androstane receptor, steroid

## Abstract

Nuclear receptors (NRs) are ligand-activated transcription factors that regulate the expression of their target genes. NRs play important roles in many human diseases, including metabolic diseases and cancer, and are therefore a key class of therapeutic targets. Steroids play important roles in regulating nuclear receptors; in addition to being ligands of steroid receptors, steroids (and their metabolites) also regulate other NRs, such as the pregnane X receptor and constitutive androstane receptor (termed xenobiotic receptors), which participate in steroid metabolism. Xenobiotic receptors have promiscuous ligand-binding properties, and their structurally diverse ligands include steroids and their metabolites. Therefore, steroids, their metabolism and metabolites, xenobiotic receptors, steroid receptors, and the respective signaling pathways they regulate have functional interactions. This review discusses these functional interactions and their implications for activities mediated by steroid receptors and xenobiotic receptors, focusing on steroids that modulate pathways involving the pregnane X receptor and constitutive androstane receptor. The emphasis of the review is on structure-function studies of xenobiotic receptors bound to steroid ligands.

## 1. Nuclear Receptors and Steroid Ligands

Nuclear receptors (NRs) function as transcription factors. When activated by binding to their ligands, NRs bind to DNA promoter regions to activate the expression of target genes [1]. NRs play important roles in signal transduction pathways; they are also associated with many human diseases and therefore are therapeutic drug targets [2]. Small molecules, including steroids, are well known NR ligands. NRs are very similar in structure, although they differ in target gene recognition and ligand specificity [3,4]. Among the different structural elements of NRs, the C-terminal ligand-binding domain (LBD), which is important for the binding of small molecules such as steroids, is the region of interest for small molecule–based drug discovery. The LBD contains the activation function-2 (AF2) domain, which is considered a key regulatory element. The most highly conserved sequence region of NRs is the DNA-binding domain (DBD), which consists of 70 amino acid residues and zinc-finger motifs that are crucial for DNA recognition and transcription [5]. Most NRs have also been found to contain an activation function-1 (AF-1) domain at their N-terminus. NRs can form homodimers or hetero-dimerize with the retinoid X receptor (RXR) through their DBDs and LBDs. 

NRs bind to diverse ligands, including steroids. Small lipophilic molecules, such as steroids and thyroid hormones, are significant in the growth, differentiation, metabolism, reproduction, and morphogenesis of higher organisms and humans. Most of these molecules’ cellular actions are mediated through binding to NRs [5]. Thus, the activity of NRs can be modulated by small molecules, and NRs can be used successfully as drug targets [2,6,7]. Small molecules such as steroid ligands play an important role in modulating NR signaling pathways; therefore, further investigation of steroid ligands and their interaction with specific NRs will have pharmacological and therapeutic relevance. Steroid ligands can modulate the estrogen receptor (ER), which is a NR superfamily member and has important roles in diverse physiological pathways. One such steroid ligand is estradiol, which activates ERα and is used to treat a variety of diseases, including menopausal symptoms (e.g., hot flashes), breast cancer, and osteoporosis [8,9,10,11]. Interestingly, previous studies have demonstrated that estradiol can also modulate xenobiotic receptor activity. This review focuses on the xenobiotic nuclear receptors pregnane X receptor (PXR) and constitutive androstane receptor (CAR), summarizing advances in the discovery of steroid ligands that modulate their activity and structure-function studies of these receptors bound to steroids.

## 2. Overview of PXR and CAR

PXR and CAR belong to the NR superfamily and are known as xenobiotic receptors. These receptors’ ligand-binding sites are promiscuous and can bind to structurally diverse compounds including exogenous and endogenous ligands. Among the NRs, PXR (NR1I2) is most closely related to CAR (NR1I3). Like other NRs, PXR and CAR have N-terminal DBD, Hinge region, and C-terminal LBD regions and these regions participate in the formation of an interacting functional domain that may be independent or allosteric [3,5,12,13,14]. PXR and CAR share ~70% amino acid identity in their LBDs and have common target genes [15,16]. Like PXR, CAR can be activated by a diverse set of steroids and can control the expression of a large number of target proteins [17,18]. Depending on the type of ligand, both PXR and CAR interact with co-activators through the ligand-dependent AF-2 helix and helix 3–5 in their LBDs [12,19]. Agonist ligands cause co-activator binding to these receptors, resulting in receptor activation, whereas antagonist ligands cause co-repressor binding that deactivates the receptors [12]. These receptors specifically recognize the L-X-X-L-L motifs (X = any other amino acid) in co-activators and the I/L-X-X-I/V-I motifs in co-repressors [12]. Binding of agonists to the LBD causes a conformational change that exposes a hydrophobic surface for co-activator binding [12]. 

## 3. Biological Processes Regulated by PXR

As a promiscuous NR, PXR binds to a broad range of structurally diverse compounds, including drugs, natural and synthetic steroids, and hyperforin in St. John’s wort [19,20,21,22], thereby regulating genes involved in drug metabolism, transport, and clearance [23]. Human PXR (hPXR) regulates the transactivation of multiple drug-metabolizing genes, including those encoding *CYP3A4*, *CYP2B6*, *UGT1A1*, and drug transporters such as multidrug resistance protein 1 (MDR1) [19,24]. PXR is involved not only in the elimination of harmful chemicals but also in many physiological disorders: bone disorders, hepatic steatosis, inflammatory bowel disease, and cancer [19,25,26]. The structures of the apo-form and ligand-bound form of PXR are very similar, unlike those of most promiscuous proteins, which generally adapt their shapes to different ligands [27]. PXR is known to be highly expressed in the liver, small intestine, and colon [20,21,22]. Some bile acids, such as lithocholic acid, can function as ligands for both human and mouse PXR. Interestingly, PXR plays an important role in the detoxification of bile acids and consequently in cholestatic disorders. Therefore, PXR and bile acid regulation are linked [20,28,29]. 

## 4. Steroids as PXR Ligands

**Figure 1:**
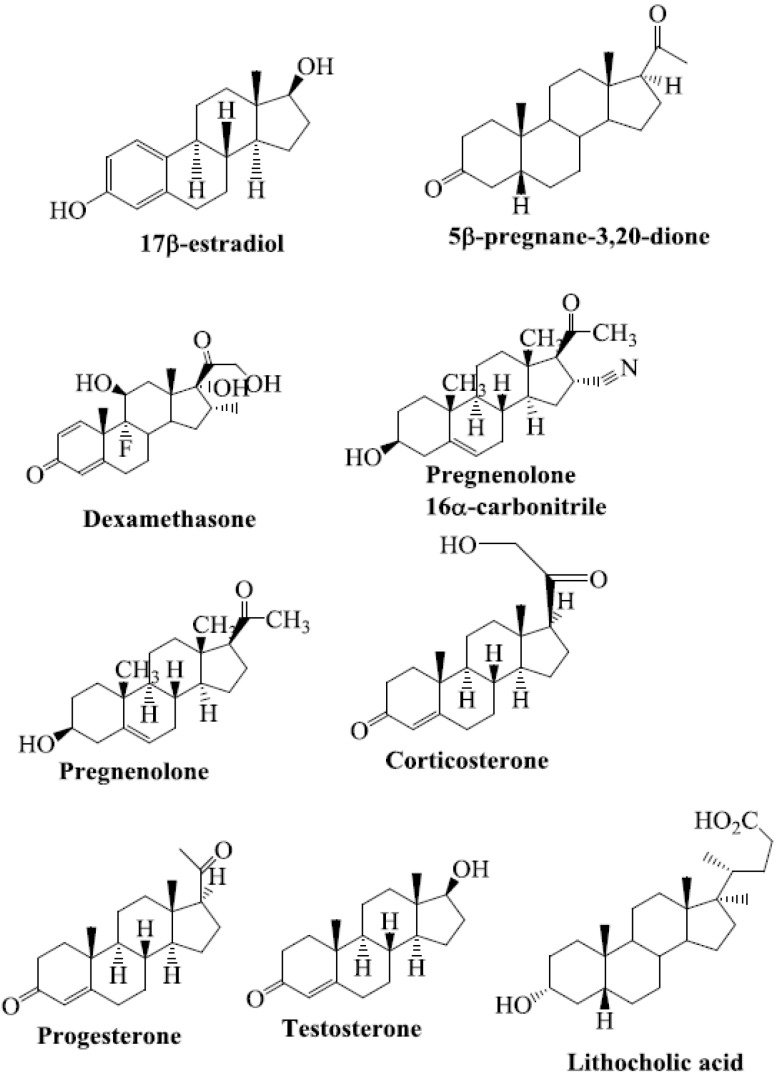
Chemical structures of selected steroid ligands known to activate PXR.

Steroids are among the structurally diverse small molecules that bind to PXR. Numerous steroid ligands bind to and modulate PXR. The key endogenous steroid ligands of PXR are 5-β-pregnane-3,20-dione, progesterones, pregnenolones, corticosterones, testosterone, the steroid-like compound dexamethasone, bile acids, and 17β-estradiol [15,21,22,30,31,32,33,34,35,36,37,38,39,40] (Figure 1). All of these steroids have been shown to regulate the function of PXR.

**Figure 2:**
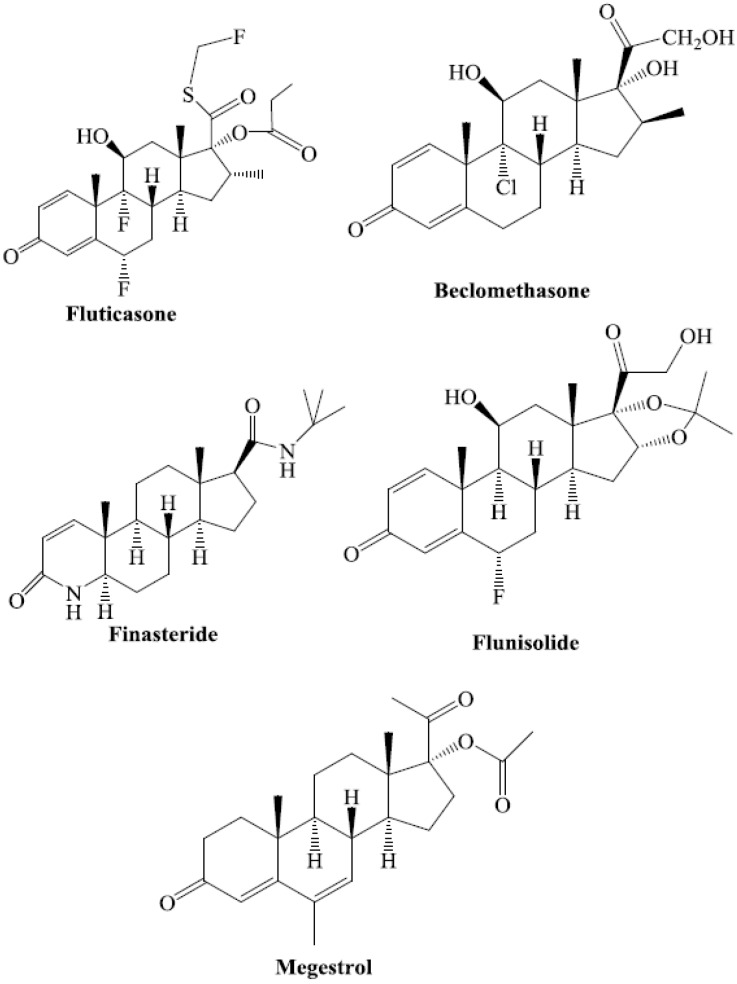
Chemical structures of FDA-approved prescription drugs that contain a steroidal moiety and are known to activate hPXR.

Exogenous steroids reported to activate PXR include fluticasone, nimodipine, nisoldipine, beclomethasone, megestrol, finasteride, and flunisolide [24] (Figure 2). These steroid ligands are U.S. Food and Drug Administration (FDA)-approved prescription drugs [24]. 

Several marine-derived natural steroid ligands are also reported to activate PXR [41]. These compounds are theonellasterols and conicasterols isolated from *Theonella swinhoei* [42]. They comprise 10 new polyoxygenated steroids, termed theonellasterols B-H and conicasterols B-D [41]. 

**Figure 3:**
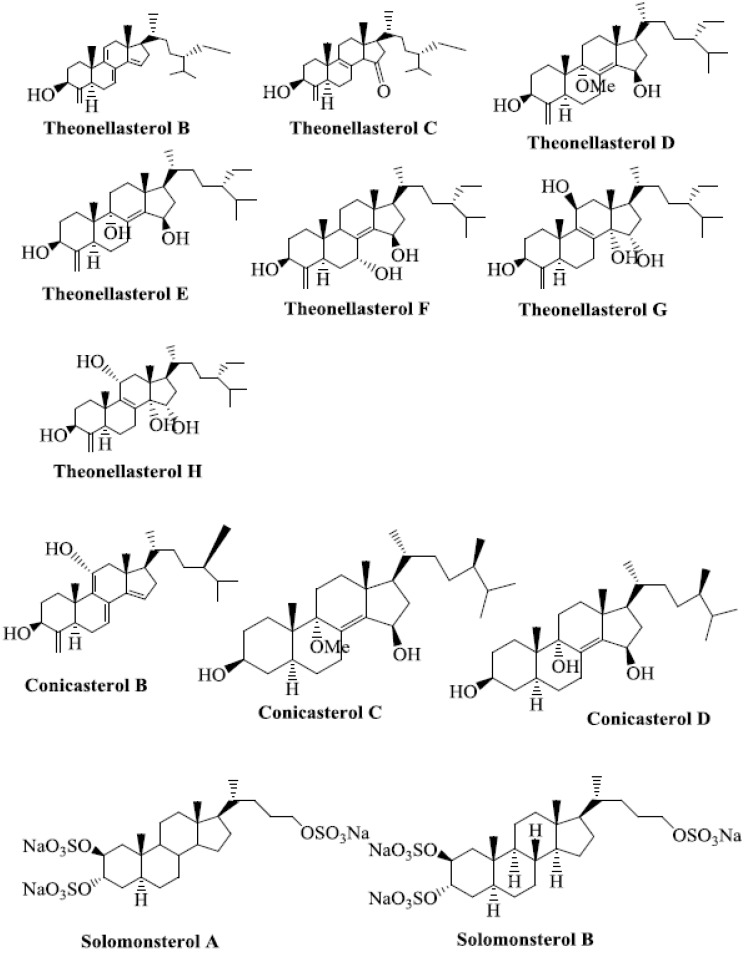
Marine-derived natural steroid ligands reported to activate PXR.

Theonellasterol H has the same molecular formula as theonellasterol G but differs in ring C [41]. Theonellasterol G has been identified as the first PXR agonist of marine origin that has potential utility in treating liver disorders [41]. Conicasterol E is a PXR agonist [43]. Fractionation of conicasterol E extract produced various steroids that are ligands of PXR. Solomonsterols A and B are two potent PXR agonists that can be used to treat immune-driven inflammatory bowel diseases [41,44,45,46] (Figure 3). 

## 5. Molecular Mechanism of PXR-Steroid Binding

**Figure 4:**
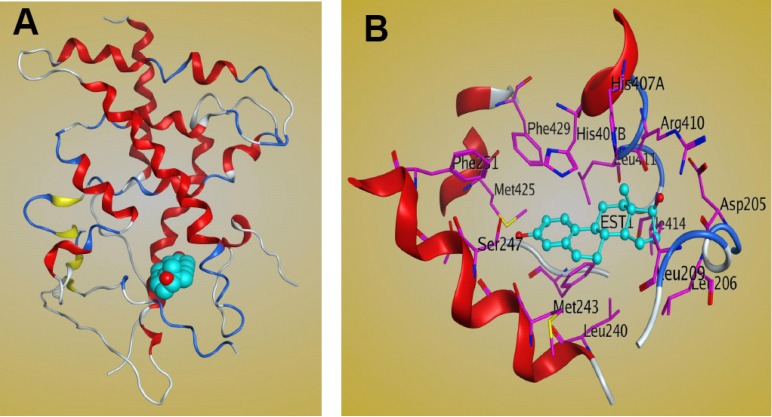
Structure of the PXR LBD–17β estradiol complex. (**A**) Ribbon representation of the PXR LBD bound to the 17β estradiol ligand (cyan). (**B**) Interactions between the PXR LBD and 17β estradiol within the ligand-binding pocket. The figure was generated by MOE software.

The LBD of PXR contains three sets of α-helices and a layer of 5-strand, anti-parallel β-sheets. The sets of α-helices include α1/α3, α4/α5/α8/α9, and α7/α10 [19,47]. The crystal structure of full-length PXR is not available, but those of the PXR LBD in both the apo- and ligand-bound forms have been solved. PXR has a well-defined binding site composed mainly of hydrophobic amino acid residues, including a few that are polar and charged. The LBD of PXR differs across species and is bound by different ligands [47]. The PXR residues that are important for ligand binding in different species have been investigated. Four residues are important for ligand contact with PXR, and these residues differentiate mouse and human PXR specificity [47]. Of the available crystal structures of PXR-LBD-ligand complexes, only one involves the steroid ligand 17β-estradiol [30] (Figure 4). Structural analysis of the complex shows that estradiol occupies only one region of PXR’s large ligand-binding pocket, leaving 1,000 Å^3^ of space unoccupied [30]. A more detailed structure shows that polar residues such as Ser247 and Arg410 form H-bonds with PXR’s LBD. The steroid A-ring forms a hydrogen bond with Ser247, while the 17β-hydroxyl group on the D-ring of the steroid forms a hydrogen bond with Arg410 [30]. Nonpolar residues also play an important role in stabilizing estradiol bonds within the LBD of PXR [30], in which hydrophobic amino acid residues, such as Met243, Leu411, and His407, form van der Waals interactions with the estradiol molecule [30]. The other two residues, Met425 and Phe429, on αAF of the PXR AF-2 surface are also involved in the interaction and stabilize the active AF-2 conformation of the receptor [30]. Additional residues that contribute to binding are Phe251, Asp205, and Ser208 [30]. The PXR-estradiol complex structure can be useful not only for understanding the mechanism of steroid binding to PXR-LBD but also for the development of steroidal modulators of PXR for clinical use.

## 6. Biological Processes Regulated by CAR

Like PXR, CAR is a promiscuous NR that binds to diverse ligands, including both exogenous and endogenous steroids. CAR belongs to a family of NRs involved in cellular development; homeostasis; drug, lipid, and energy metabolism; detoxification; and clearance [48,49]. Moreover, CAR acts as a chemical sensor of xenobiotics, endogenous compounds, and toxic metabolic by-products that modulate CAR-mediated transcription of genes involved in the oxidation and elimination of these compounds [50,51]. To induce gene transcription, CAR forms a heterodimer with RXR that binds to promoters and induces the expression of target genes encoding phase I (*CYP2B6*) and phase II (*UGT1A1*, *GSTA1* and *GSTA2* ) enzymes and phase III drug transporters (*SLC21A6*, *ABCC2*) [52,53,54,55]. Both CAR and PXR have been implicated in regulating the expression of *CYP2B*-, *CYP2C9*-, and *CYP3A*-family enzymes [56,57,58,59,60,61,62]. CAR is expressed most abundantly in liver and intestine, and unlike other NRs it has strong constitutive activity in the absence of its ligands [63,64,65]. Previous studies have demonstrated the repressive effects of androstenol on CAR constitutive activity via inverse agonism [66]. CAR is also reported to have physiological roles in cholesterol elimination, bilirubin clearance, and homeostasis of circulating thyroid hormones, as well as implications for metabolic disorders and other human diseases [53,67,68,69,70]. Because the activity of CAR can be modulated by small-molecule ligands, CAR may be a potential novel target for drug development. However, modulation of CAR activity is complicated by CAR’s ability to metabolize parent drug compounds to toxic metabolites that exert deleterious effects [71].

## 7. Steroids as CAR Ligands

**Figure 5:**
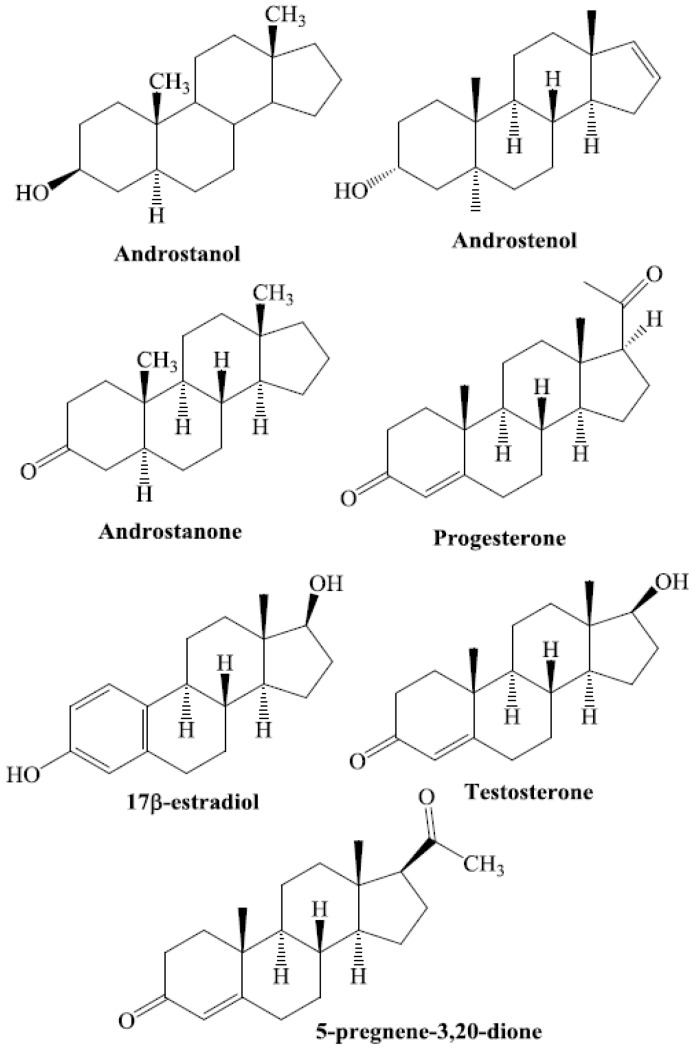
Chemical structures of selected steroid ligands known to activate CAR.

CAR exhibits both structurally diverse and species-specific ligand binding properties. The promiscuous ligand binding of CAR, and the alteration of CAR activity by fluctuating levels of endogenous compounds such as steroids, add a layer of complexity to understanding the mechanisms by which CAR regulates various biological processes [72,73,74,75,76,77,78,79]. Steroid ligands such as estrogen (17β-estradiol), progesterone, and androgens have been shown to modulate CAR activity (Figure 5). 

Kawamoto and colleagues found that 17β-estradiol and estrone, but not other synthetic estrogen derivatives (estriol, estretrol, estradiol sulfate, and synthetic estrogen diethylstilbestrol), induced CAR activity, as assessed by activation of the NR1 enhancer (a response element found in the *CYP2B6* gene) [72]. Further, they found that progesterone and androgens suppressed NR1 activity in HepG2 cells. Similar results were seen in mouse primary hepatocytes and transient expression of CAR in rat HepG2 cells. Interestingly, neither agonistic nor antagonistic effects were seen when human CAR (hCAR) was transiently expressed in HepG2 cells, demonstrating the species-specificity of CAR ligand responsiveness [72].

## 7. Structural Investigation of Interactions between CAR and Its Steroid Ligands

CAR contains a DBD in its N-terminal region, followed by the hinge domain and the C-terminal LBD, which is also an important interface for dimerization with the RXR [51,80,81]. The crystal structure of CAR in complex with an agonist has been reported [82]. The crystal structure of the LBD of CAR revealed that conformational changes occur upon ligand activation. It is well documented that the C-terminal helix H12 is important and contributes to the formation of AF2. Helix H12 is firmly positioned at the top of the ligand-binding pocket, where it contributes to the interaction surface for CAR co-activators. Upon binding, co-activators can recruit additional mediating factors to form a pre-initiation complex. The CAR co-activators identified to date include glucocorticoid receptor-interacting protein 1 (GRIP1/TIF2), PGC-1, SRC-1, SMC-1, and ASC-2 [51,83,84,85,86,87,88]. Conversely, antagonists inhibit the active conformation, thus providing an interface for co-repressor binding to CAR’s LBD and repressing gene expression. Several reports describe steroidal modulation of CAR activation. Jyrkkarinne and colleagues, using a 3D-QSAR analysis based on the GAL4-mCAR LBD fusion protein, demonstrated the inhibition of mouse CAR (mCAR) by more than 40 steroids, including 5β-pregnane-3,20-dione and 5α-androstan-3α-ol. They found that steroids could inhibit mCAR, while a large group of diverse chemicals, including estrogen, could act as mCAR agonists [89]. One of the steroid ligands, 17β-estradiol, was shown to act as an agonist of mCAR but as an antagonist of human CAR, again demonstrating the differential species-dependent responsiveness of CAR to diverse ligands. Differences between the amino acid residues of the ligand-binding pocket (Ser251, Ile252, and Leu253 of mCAR and Phe243 of hCAR) significantly affected the responsiveness of CAR to 17β-estradiol [75]. More specifically, 17β-estradiol recruited the co-regulators SRC-1 and nuclear receptor co-repressor NCoR when bound to mouse CAR [75], but not when bound to hCAR [74]. Repo and colleagues identified specific amino acid residues crucial for basal mCAR activity. In addition, mutation of the Phe171 and Tyr336 residues within the ligand-binding pocket produced differential effects on ligand-specific activity. Another steroid ligand shown to bind to CAR and modulate its basal activity is 5α-androst-16-en-3α-ol (androstenol). Forman and colleagues reported that androstenol suppressed CAR activity upon binding, via inverse antagonism [66]. Specifically, Shan demonstrated conformational disruption of AF-2, inducing the recruitment of NCoR [81]. Other studies demonstrated that Phe171 is a residue crucial for NCoR recruitment and the responsiveness of mCAR to androstenol. Moreover, Arg175 was shown to be essential for androstenol-mediated inhibition [75]. Interestingly, in humans the Arg175Ala substitution enhanced androstenol-mediated recruitment of NCoR to CAR, suggesting the significance of species differences in ligand specificity [75].

**Figure 6:**
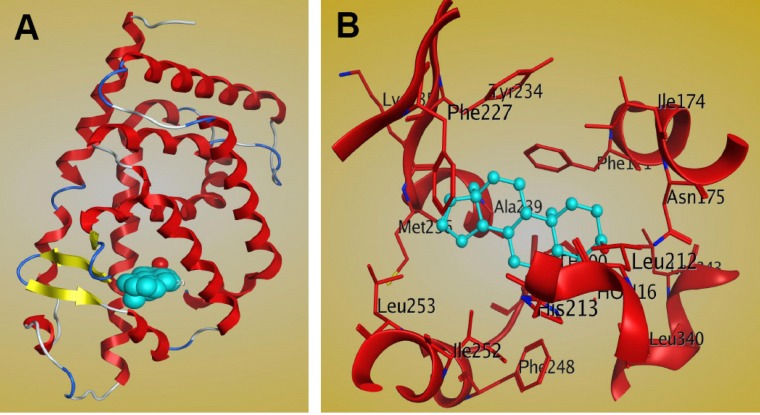
Structure of the mCAR LBD–androstenol complex (PDB: 1XNX). (**A**) Ribbon representation of one of the two CAR molecules in the asymmetric unit bound to the ligand androstenol (cyan). (**B**) Interactions between CAR and androstenol within the ligand binding pocket, as shown by MOE software.

The complex structure of the mCAR LBD bound to the steroidal ligand androstenol has been reported (PDB: 1XNX) [81]. It shows that the crystallographic asymmetric unit comprises two molecules and that the main conformational difference between the molecules lies within the region connecting helices H1 and H3 (residues 156–163). These regional differences cause the two molecules to assume different conformations, and in the absence of these residues the two molecules are identical [81]. The binding cavity of the CAR LBD, with a volume of 525–675 Å^3^ [80,81,82], is smaller than that of PXR, which is greater than 1,100Å^3^ [47]. The CAR LBD consists of 12 α-helices and three β-strands [81]. The 3_10_ helices found in the LBD may be important for gate formation for ligand binding, and may contribute to the hydrophobic nature of the CAR ligand-binding pocket [81]. There are two 3_10_ helices between H1 and H3 and an additional helix between H3 and H4, which are uncharacteristic of CAR structure [81]. Structural analysis of the CAR LBD showed that the binding cavity is composed mainly of apolar residues, including Phe142, Phe171, Ala172, Ile174, Met178, Val209, Leu212, Leu216, Phe 220, Phe227, Cys229, Tyr234, Met236, Ala239, Phe244, Phe248, Leu249, Ile252, Leu253, Leu340, Asn175, His213, Glu225, Asn226, Lys235, Asp238, and His256 [81]. The complex structure of mCAR-androstenol showed that androstenol interacts specifically with the hydrophobic ligand binding pocket of the CAR LBD (Figure 6). Androstenol forms hydrogen bonds and interacts with apolar residues in H3, H5, H6, H7, H11, and the β strand β2 [81]. Two polar interactions between the 3α-hydroxyl moiety of androstenol and the CAR residues Asn175 of helix H3 and His213 of helix H5 are observed, and these interactions are similar in both CAR molecules in the asymmetric crystallographic unit [81]. CAR residues that interact with androstenol are shown in Figure 6B. However, mutagenesis and cell-based transactivation assays show that the apolar contributions of Phe171 and Ile174 contribute to androstenol binding, while Met236 is not essential [81]. 

**Figure 7:**
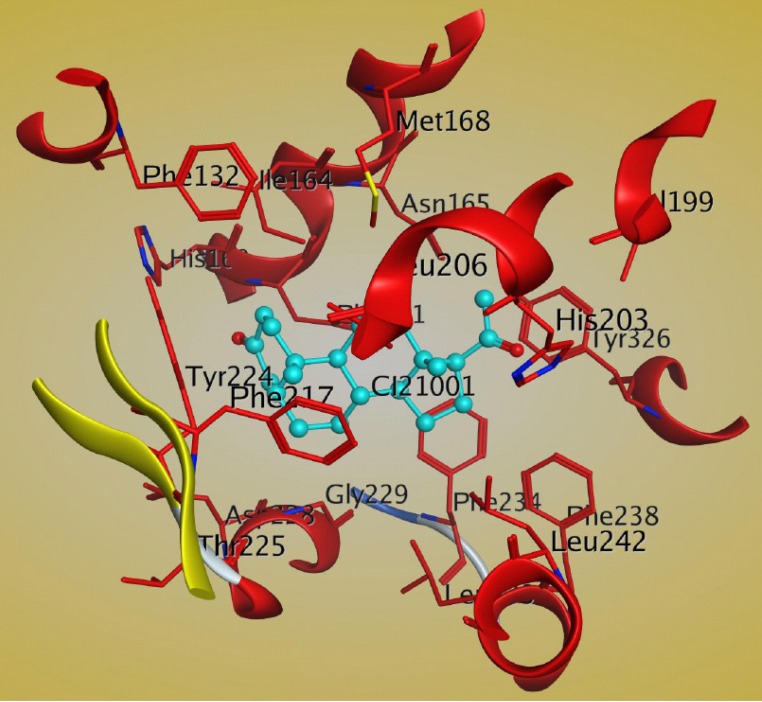
Structural analysis of the interactions between mCAR LBD and 5β-pregnane-3,20-dione (cyan) within the ligand binding pocket, as shown by MOE software (PDB:1XV9). The important residues of CAR are shown in red. The figure was generated by using MOE software.

There is another report of the crystal structure of the mCAR-LBD in complex with the steroid ligand 5β-pregnane 3, 20-dione (PDB: 1XV9) [80]. The steroid molecule interacts with the hydrophobic residues Phe161, Ile164, Leu206, Phe217, Tyr224, Phe234, and Leu242 of CAR [80]. There is a hydrogen bond between the C21 ketone and His203 that may help to stabilize the steroid molecule in the ligand-binding pocket of CAR [80]. The important residues of the CAR LBD that bind to 5β-pregnane 3, 20-dione are shown in Figure 7. 

This CAR-steroidal ligand complex structure will be useful in elucidating how steroid ligands bind to the CAR LBD and in structure-based drug design.

## 8. Conclusions

Steroids are involved in regulating multiple biological processes, including those mediated by NRs, and have pharmacological and therapeutic significance. PXR has evolved to detect structurally diverse sets of compounds including exogenous drugs and toxins as well as endogenous compounds which are uncommon for other nuclear receptors such as steroid, retinoid, and thyroid hormone receptors [22,31,47]. The physical binding of steroid ligands has been found to play an important role in modulating PXR and CAR activity. There are several reports on the affinities of PXR and CAR to their steroid ligands. The affinities are mostly in the range of low-micromolar to sub-micromolar. It has been reported that lithocholic acid which is an agonist for PXR showed EC_50_ of 9 µM [20]. However, 17β-estradiol showed EC_50_ of 22 µM for PXR [30]. Other reports showed that solomonsterol A has EC_50_ of 0.4 µM for binding to PXR [45]. For dexamethasone and androstanol, EC_50_ values are >10 µM [15]. CAR also showed similar trend in terms of affinities to steroids. It has been reported that metabolites of androstane 5α-androst-16-en-3α-ol and 5α-androstan-3α-ol bind to CAR with IC_50_ values about 0.4 µM [66]. There are other reports of steroids binding to CAR such as testosterone and progesterone which showed IC_50_ values of 35 and 11 µM respectively [89]. Some steroids, such as 17β-estradiol, 5β-pregnane-3,20-dione, and clotrimazole, can modulate both PXR and CAR, possibly because of similarity in the receptors’ ligand-binding pockets. It has also been found that steroids have different effects on PXR and CAR. One such example is androstanol, which is an agonist for PXR but an antagonist for CAR [90]. The effects of steroids on the xenobiotic receptors PXR and CAR indicate that the biological functions of steroids exceed those mediated by steroid receptors. It has been reported that some steroids activate PXR and CAR, though EC_50_ values are well-above their physiological levels. Therefore, it is possible that the steroids are not real endogenous ligands for PXR or CAR. The EC_50_ values of E2 for hPXR and estrogen receptors are significantly different. This can be explained from structural point of view. E2 binds in the LBD of PXR adjacent to the αAF helix that causes 1,000 Å^3^ of LBD space unoccupied. On the contrary, E2 occupies almost the entire ligand binding pocket of estrogen receptor that causes E2 to show strong binding that reflects in EC_50_ for ERα (1 nM) compared to hPXR (22 µM). The physiological level of the hormone *in vivo* is 1 nM which is far below the EC_50_ for hPXR. The very low affinity of E2 for hPXR explains the negligible physiological role of E2 on hPXR action [12,30,91,92]. Testosterone in healthy adult males has been shown to vary between 315 and 1,000 ng/dL (11 and 35 nmol/liter) [93]. Jyrkkarinne and colleagues reported various steroids, including androstane metabolites involved in the biosynthesis of testosterone, as inhibitors of CAR activity. However, the estimated pIC_50_ of the various steroids were in the micromolar range, which questions the physiological relevance of the inhibitory mechanism reported. Interestingly, when these data were reported, there are no clear structural requirements for mCAR ligands, a defined mechanism of action, nor any clear quantitative structure activity analysis data for mCAR activators or inhibitors [89]. Although these concentrations may not be physiologically relevant, the compounds reported define a class of novel compounds that provide structural relevance and can contribute to the discovery of a class of structurally similar steroid-like compounds that may potentially act as CAR antagonists [89]. Nevertheless, by developing a 3D-QSAR model for mCAR, the ligand-mediated inhibitory mechanism of action has been defined with clarification of ligand specificity and identification of regions specific for the steroid sensitivity of mCAR, which can contribute to novel CAR-based drug discovery [89]. Some steroidal compounds are used as drugs, and PXR and CAR regulate drug metabolism. Therefore, further investigation and mechanistic analysis of the interactions between PXR/CAR and their steroid ligands will provide fundamentally important information and a better understanding of steroid-mediated physiological responses. 
